# Bovine Colostrum Supplementation During Running Training Increases Intestinal Permeability

**DOI:** 10.3390/nu1020224

**Published:** 2009-12-02

**Authors:** Jonathan D. Buckley, Ross N. Butler, Emma Southcott, Grant D. Brinkworth

**Affiliations:** 1 Nutritional Physiology Research Centre, University of South Australia Adelaide, South Australia, 5000, Australia; 2 Sansom Institute for Health Research, University of South Australia Adelaide, South Australia, 5000, Australia; 3 Department of Gastroenterology, Adelaide Women’s and Children’s Hospital, Adelaide, South Australia, 5000, Australia; 4 Preventative Health National Research Flagship, Commonwealth Scientific and Industrial Research Organisation – Human Nutrition, Adelaide, South Australia, 5000, Australia

**Keywords:** intestinal transport, exercise, gut permeability, lactulose, rhamnose

## Abstract

Endurance exercise training can increase intestinal permeability which may contribute to the development of gastrointestinal symptoms in some athletes. Bovine colostrum (BC) supplementation reduces intestinal permeability induced by non-steroidal anti-inflammatory drugs. This study aimed to determine whether BC could also reduce intestinal permeability induced by endurance exercise. Thirty healthy adult males (25.0 ± 4.7 yr; mean ± SD) completed eight weeks of running three times per week for 45 minutes at their lactate threshold while consuming 60 g/day of BC, whey protein (WP) or control (CON). Intestinal permeability was assessed at baseline and after eight weeks by measuring the ratio of urinary lactulose (L) and rhamnose (R) excretion. After eight weeks the L/R ratio increased significantly more in volunteers consuming BC (251 ± 140%) compared with WP (21 ± 35%, P < 0.05) and CON (−7 ± 13%, P < 0.02). The increase in intestinal permeability with BC may have been due to BC inducing greater leakiness of tight junctions between enterocytes or by increasing macromolecular transport as it does in neonatal gut. Further research should investigate the potential for BC to increase intestinal macromolecular transport in adults.

## 1. Introduction

Supplementation with bovine colostrum (BC) during athletic training improves exercise performance for both endurance [1] and anaerobic [2,3] activities. BC has also been shown to improve recovery [4] and buffer capacity during exercise [5], as well as increasing resistance to upper respiratory tract infection [6].

The epithelial lining of the intestines constitutes a selectively permeable barrier which permits the absorption of nutrients, electrolytes, and water, while excluding the absorption of intraluminal pathogens and other potentially harmful molecules. Endurance exercise can increase the permeability of the intestinal barrier [7,8] and this might contribute to some of the gastrointestinal symptoms that are evident in endurance athletes after prolonged exercise [9]. BC supplementation has been shown to reduce the intestinal permeability that is induced by the consumption of non-steroidal anti-inflammatory drugs [10], and might therefore also be useful for reducing exercise-induced intestinal permeability. Thus, given that BC is a supplement consumed by endurance athletes to improve performance and recovery, we hypothesized that it might also reduce the intestinal permeability that can be induced through the performance of endurance exercise, and therefore potentially protect against the development of endurance exercise-induced gastrointestinal disturbances.

The purpose of the present study was to determine whether, compared with whey protein (WP) or a non-supplemented control group (CON), an eight week period of supplementation with BC during regular endurance exercise training could protect against an increase in exercise-induced intestinal permeability.

## 2. Results and Discussion

### 2.1. Age, Height and Mass

There were no differences in age (BC 26.7 ± 1.5 yr, WP 25.4 ± 1.6 yr, CON 23.7 ± 1.3 yr; P = 0.33), height (BC 181.3 ± 2.0 cm, WP 177.5 ± 1.9 cm, CON 179.5 ± 2.3 cm; P = 0.55) or body mass (BC 78.2 ± 3.2 kg, WP 78.6 ± 3.9 kg, CON 76.6 ± 2.7 kg; P = 0.88) between the groups at Week 0. Neither height (P = 0.57) nor body mass (P = 0.24) changed significantly in any group during the study period.

### 2.2. Exercise Parameters

#### Peak oxygen uptake (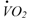)

There was no difference in peak oxygen uptake (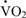) between groups at Week 0 (BC 52.8 ± 1.6 mL·kg^-1^·min^-1^, WP 53.1 ± 2.0 mL·kg^-1^·min^-1^, CON 57.3 ± 3.0 mL·kg^-1^·min^-1^; P = 0.15). While peak 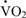 tended to increase in all groups (BC 0.6 ± 1.3 mL·kg^-1^·min^-1^, WP 0.2 ± 1.6 ml·kg^-1^·min^-1^, CON 2.2 ± 1.0 mL·kg^-1^·min^-1^), this did not reach statistical significance (P = 0.22). 

#### Exercise duration

There was no difference in the running time to exhaustion (TTE) between groups at Week 0 (BC 29.1 ± 2.8 min, WP 29.1 ± 3.6 min, CON 32.3 ± 1.7 min; P = 0.56). TTE had increased in all groups by Week 8 (P < 0.001), reaching 32.7 ± 2.4 min in BC, 31.7 ± 3.2 min in WP, and 33.6 ± 1.7 min in CON, but the magnitude of these increases did not differ between groups (P = 0.46).

#### Peak Heart Rate (HR)

There was no difference in peak heart rate (HR) between groups at Week 0 (BC 194 ± 4 beats·min^-1^, WP 192 ± 3 beats·min^-1^, CON 194 ± 2 beats·min^-1^; P = 0.84). Peak HR had decreased in all groups by Week 8 (P < 0.001), reaching 189 ± 4 beats·min^-1^ in BC, 188 ± 3 beats·min^-1^ in WP, and 193 ± 2 beats·min^-1^ in CON. The magnitude of these decreases did not differ between groups (P = 0.06) despite a tendency for greater reductions in BC and WP.

#### HR at lactate threshold (i.e. training HR [HR_t_])

There was no difference in HR_t_ between groups at Week 0 (BC 165 ± 4 beats·min^-1^, WP 164 ± 5 beats·min^-1^, CON 159 ± 4 beats·min^-1^; P = 0.56). HR_t_ had decreased in all groups by Week 4 (P = 0.02), but there was no difference in the magnitude of decrease between groups (P = 0.75), reaching 162 ± 2 beats·min^-1^ in BC, 156 ± 4 beats·min^-1^ in WP, and 154 ± 4 beats·min^-1^ in CON. There was no further change in HR_t_ between Weeks 4 and 8 (P = 0.80).

### 2.3. Dietary Intake

There was no difference in mean daily energy intake between groups over the study period (BC 9231 ± 484 kJ, WP 10366 ± 673 kJ, CON 9511 ± 612 kJ; P = 0.46). Nor was there any difference in the contribution of carbohydrate as a percentage of dietary intake (BC 49.6 ± 2.0 %, WP 48.1 ± 3.6 %, CON 48.1 ± 2.2 %; P = 0.90). The consumption of protein by the BC and WP groups constituted a significantly greater proportion of the dietary intake than in the CON group when expressed as either an absolute mean daily intake (BC 135.6 ± 7.1 g, WP 140.7 ± 9.6 g, CON 105.3 ± 7.5 g; P < 0.02), or as a percentage of mean daily energy intake (BC 24.7 ± 0.7 %, WP 22.9 ± 1.0 %, CON 19.9 ± 0.9 %; P < 0.03). There was a tendency for the CON group to consume more fat as a percentage of dietary intake, although this did not reach statistical significance (BC 24.4 ± 1.4 %, WP 26.9 ± 3.2 %, CON 31.3 ± 2.1 %; P = 0.08).

### 2.4. Intestinal Permeability

**Figure 1:**
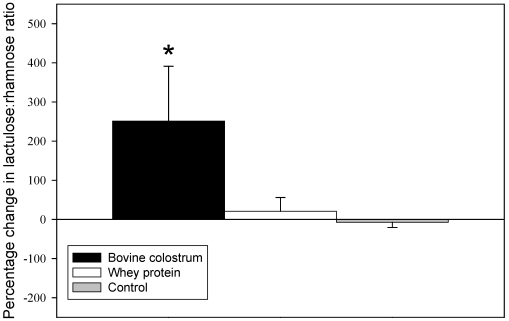
Percentage change from baseline in lactulose/rhamnose ratio following eight weeks of endurance exercise training and supplementation with 60 g·day^-1^ of concentrated bovine colostrum protein powder, concentrated whey protein powder, or no supplement (Control). * Significantly different from whey protein (P < 0.05) and control (P < 0.02).

At baseline the lactulose to rhamnose (L/R) ratio was significantly higher in the WP group compared with the other two groups (BC 0.06 ± 0.01, WP 0.14 ± 03, CON 0.05 ± 0.01; P < 0.02). There was no significant difference between BC and CON (P = 0.55). Normalising for changes in the L/R ratio over the eight week study period by expressing them as percentage changes, and using the values at baseline as a covariate, analysis of covariance (ANCOVA) revealed that supplementation with BC had resulted in a significantly greater increase in the L/R ratio compared with WP (P < 0.05) and CON (P < 0.02)(Figure 1). The changes in WP and CON were not significantly different from each other (P = 0.79). No participants reported any adverse gastrointestinal symptoms.

The principal finding of the present study was that supplementation with BC during eight weeks of endurance running training significantly increased intestinal permeability. This effect did not appear to be related to the running training itself since neither the control group nor the WP group, which also exercised, exhibited any significant changes in permeability. While all three groups improved their treadmill running time to exhaustion, indicating that they had improved their fitness, there was no greater improvement in the group which consumed the BC which is consistent with a previous finding from our laboratory [4].

The lack of increase in intestinal permeability due to exercise might reflect an insufficiency of intensity or duration of the exercise training program, particularly since the program was not sufficient to increase peak 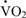. Nevertheless, the increase in intestinal permeability with BC supplementation was a somewhat unexpected finding given that BC has been shown to attenuate the intestinal hyperpermeability induced by non-steroidal anti-inflammatory drugs (NSAIDs) [11]. The finding of an increase in permeability suggests that in healthy adult gut BC might induce an opposite effect and increase the leakiness of tight junctions between enterocytes, although no participants reported any adverse gastrointestinal symptoms. Alternatively, this finding could be interpreted as indicating that BC increased the intestinal transport of larger molecules. Colostrum contains components which facilitate intestinal macromolecular transport in neonates prior to gut closure [12,13], with the increased transport being ascribed to colostral proteinase inhibitors protecting against molecular digestion and bioactive components stimulating transport pathways [12,13]. The lactulose/rhamnose test of intestinal permeability employed in the current study is based on the assumption that rhamnose, a relatively small monosaccharide (164 Da), will permeate the epithelia transcellulary, while the larger lactulose disaccharide molecule (342 Da) will only be absorbed paracellulary through the tight junctions between the epithelial cells [14]. The test is not able to specifically determine the pathway by which lactulose is transported across the intestinal epithelium and it is possible that BC increased paracellular transport by increasing the gaps between the tight junctions of enteroendocytes, but it also cannot be ruled out that BC may have increased intestinal transport by endocytosis or some other mechanism, as it does in neonatal animals. If this were the case then some of the bioactive components in BC which promote macromolecular transport in the neonatal gut [12] might also facilitate the transport of larger molecules in the adult human gut.

While BC contains a number of growth factors and other biologically active macromolecules that might contribute to the observed physiological effects on exercise performance and immune function it is generally accepted that the adult gut is impermeable to macromolecular transport such that even if these components survived digestion, they would not be absorbed. Indeed, Mero *et al*. [15] recently demonstrated that IGF-I, which is present in high concentrations in BC [16], was broken down during digestion and did not appear intact in the circulation in the adult gut. However, in that study recombinant IGF-I (^123^I-rhIGF-I) was consumed in conjunction with a standard breakfast rather than with colostrum and the proteinase inhibitors in colostrum [13] may protect larger proteins from degradation while other bioactive components [12] facilitate its transport. We recently examined the effect of BC on the intenstinal transport of nutrient monomers (*i.e.*, glucose and alanine) [17], and found no evidence of any effect on the intestinal absorption of these molecules. However, no studies have been carried out to determine whether BC can increase the transport of larger macromolecules in the mature adult gut, and although the present data indicated that the absorption of a larger molecule (*i.e.*, lactulose) was increased this molecule is still only a relatively small disaccharide (342 Da) and does not necessarily indicate that a similar effect will be found for larger molecules. However, if BC can increase the transport of growth factors and other large biologically active proteins, then this might provide a mechanism for some of the reported increases in circulating concentrations of these molecules [15,18] and explain some of the physiological effects of this supplement. 

## 3. Experimental Section

### 3.1. Participants

Thirty healthy young adult males, aged 25.0 ± 4.7 yr (mean ± SD) completed the requirements of this study. All of the participants had been participating in regular exercise training for at least three months prior to study commencement and were medically screened using a modified pre-exercise screening questionnaire to determine their suitability to undertake exercise testing and/ or training [19]. None of the participants had any known illness, chronic disease or were suffering from conditions likely to affect intestinal permeability (e.g., celiac disease or previous intestinal surgery) and were not using medication, other drugs or had taken any BC supplements during the three months prior to the study commencement. During the study, participants were not permitted to use any nutritional supplements or non-steroidal anti-inflammatory medication, including aspirin and were asked to restrict their alcohol consumption to no more than two standard drinks per day. The study protocol and the potential risks and benefits were fully explained to each participant before they provided written informed consent. All experimental procedures were approved by the Human Ethics Committee of the University of South Australia.

### 3.2. Experimental Protocol

The study employed a placebo-controlled, randomised, parallel design. At the start of the 8-week study period, participants completed a 5-hour urine permeability test in the morning (*i.e.*, Study Day 1). Two days later (*i.e.*, Study Day 3 of Week 0), participants attended the laboratory for assessment and thereafter at four weekly intervals (*i.e.*, Study Days 31 and 59) after a minimum four hour fast (water as required). Tests were conducted at the same time of day to avoid circadian effects. At each laboratory testing session body mass, and stature were measured prior to the performance of an incremental treadmill running test to volitional exhaustion while expired air was collected for determination of gas exchange parameters, and fingertip blood samples were taken for determination of blood lactate concentrations. On the day following the final laboratory testing session (*i.e.* Study Day 60), participants repeated the 5-hour urine permeability test in the morning.

After being tested at Week 0, thirteen participants acted as controls while the others were randomly allocated to the consumption of 60 g·day^-1^ of concentrated bovine colostrum protein powder (intact^TM^, Numico Research Australia Pty Ltd, Adelaide, Australia; n = 9) or concentrated whey protein powder (Alacen, New Zealand Milk Products Australia, Rowville, Australia; n = 8) in a double blind manner. All supplements were provided in pre-weighed 20 g sachets and the participants mixed and consumed the contents of one sachet with their morning meal, and two sachets with their evening meal. The contents of each sachet were mixed with 85 mL of warm water and 40 mL of milk, shaken vigorously, and then chilled before drinking. The taste and colour of the two supplements were indistinguishable. Participants began consuming the appropriate test supplements and commenced a 3-day per week running training program on the day following the initial treadmill laboratory testing session (*i.e.*, Study Day 4).

### 3.3. Body Mass and Height

Body mass was measured using electronic digital scales (AND Mercury, FV-150, Tokyo, Japan). Height was measured by the stretch stature method [20], using a stadiometer (SECA, Hamburg, Germany).

### 3.4. Treadmill Running Test

Baseline data were collected whilst participants stood quietly on the treadmill (Quinton Instruments, Model 1860, Bothell, WA, USA) for three minutes prior to commencing each running test. Participants then commenced running at a speed of 10 km·hr^-1^ and 0% grade. The treadmill speed remained constant throughout the test and the work load was incremented every three minutes by increasing the slope of the treadmill by 1% grade until the participant reached volitional exhaustion. Preliminary experiments indicated that participants reach exhaustion after ~30 min using this protocol. The first test was conducted two weeks after a familiarisation trial.

### 3.5. Cardiorespiratory Assessment

Measurements of oxygen uptake and carbon dioxide production were recorded as 30 second averages throughout each treadmill run and the values averaged over the final 30 sec of each work load were recorded as the measured values. Participants breathed through a low resistance respiratory valve (Hans Rudolph 2700 series, Kansas City, KS, USA) with a pre-calibrated large flow turbine transducer (P.K. Morgan Mark 2, Seaford, Australia) attached to the inspiratory port to measure ventilatory volumes. Expired air was directed to a 2.6 L mixing chamber (Sportech, Canberra, Australia) from which dried gas was sampled continuously (~ 500 ml·min^-1^) and passed to an oxygen analyser (Ametek S-3A/I, Pittsburgh, PA, USA) and a carbon dioxide analyser (Ametek CD-3A), both of which had been calibrated prior to each exercise test with commercially-produced gas mixtures of known O_2_ and CO_2_ percentages (BOC Gases, Adelaide, Australia). The electrical outputs from the ventilation meter and gas analysers were integrated using a personal computer which calculated the necessary ventilatory variables. HR was recorded as five second averages during the three minute rest period prior to each treadmill run and during each run using a Sport Tester heart rate microcomputer and chest transmitter (Polar Accurex Plus, Polar Electro, Oulu, Finland). The five second averages at the end of the three minute rest period prior to each treadmill run, at the end of each three minute work load, and at the end of exercise were taken to be the measured values for HR.

### 3.6. Blood Collection and Analysis

Blood lactate concentrations were determined from fingertip blood samples (50 μL) taken at the end of the three minute rest period prior to the treadmill run, at the end of each three minute work load during the run, and immediately upon the cessation of exercise. Twenty five μL of each sample was immediately passed through an automated lactate analyser (Yellow Springs International, Model 1500 Sport, Yellow Springs, OH, USA). The remainder of each sample was discarded.

### 3.7. Lactate Threshold

A log-log transformation [21] of the 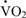 and the blood lactate concentrations during the treadmill run were used to determine the 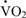 corresponding to the lactate threshold. Linear regression analysis of the HR vs 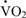 response during exercise was then used to determine the HR corresponding to the 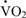 at the lactate threshold. This HR was used as the training HR (HR_t_) for the running training program.

### 3.8. Running Training Program

Participants were provided with a HR monitor (Polar Beat, Polar Electro, Finland) and ran for 45 min, three times per week on non-consecutive days at HR_t_. During the first four weeks of the study they ran at the HR_t_ determined from the testing undertaken at Week 0. During the second four weeks of the study they ran at the HR_t_ determined from the testing undertaken at Week 4.

### 3.9. Intestinal Permeability

Intestinal permeability was assessed after an overnight fast and following abstinence from alcohol consumption or ingestion of any NSAID, including aspirin, for a minimum of 48 hours. At the beginning of the test procedure, participants emptied their bladders and collected a pretest urine sample into a 30 mL sterile tube containing 25 μL of thiomersal (BDH Chemical Ltd., Poole, UK). The pretest sample was used to check any materials interfering with the peaks of the probe sugars in the high performance liquid chromatography (HPLC) assay. Within five minutes of the pre-sample collection, participants ingested the permeability test solution containing 5 g lactulose (as 7.5 mL of Duphalac^®^ syrup, Solvay-Duphar, B. V., Holland) and 1g L-rhamnose (Sigma Chemical Co., St Louis, MO, USA) dissolved in distilled water to a volume of 100 mL (Milli Q water, E. Merck, Darmstadt, Germany). Participants refrained from drinking water for the first 30 min after consuming the sugar solution. Following this initial 30-minute period, participants were encouraged to drink water as required for the next 2.5 hours of the test to ensure adequate urine output. Normal eating and drinking was allowed 3 h after ingestion of the test solution. All urinary output over five hours post sugar ingestion was collected and pooled in a large polypropylene bottle, containing 100 μL of thiomersal. If participants had not urinated in the last half an hour of the test, they were asked to collect their next void. The urine containers were kept cool during this period by placing ice packs around them in a carrying case or by placing them in a refrigerator. After the final collection, all urine samples were frozen and stored at −20 °C for subsequent analysis of lactulose and rhamnose recovery.

The total volume of the five-hour urine sample was recorded. Preparatory methods and HPLC analysis of urine for lactulose and rhamnose was conducted according to previously described methods [22]. Urine samples were initially desalted, in which 2 mL of urine was washed and mixed with 0.5 g of ion-exchange resin (Duolite MB 5113; BDH Chemical, Poole, UK). After centrifugation (10 min at 3,000 *g*) of the mixture, the supernatant was filtered through 0.2 μm (pore-size) disposable syringe filters, for analysis of lactulose and rhamnose by HPLC (Acrodisc^®^; Gelman Sciences, Ann Arbor, MI, USA). Urinary concentrations of sugar probes were calculated from the standard curves using elution peak height analysis. In each sample concentration and total urinary secretion of lactulose and rhamnose was measured. The percent excretion of lactulose and rhamnose in the urine samples was calculated as a percentage of the dose administered (*i.e.*, recovery) by converting the concentration of each sugar in the urine (μmol/L) to a mass (g) (*i.e.*, concentration x volume of urine produced) and then calculating the percentage excreted. The ratio of % lactulose excretion to % rhamnose excretion (L/R ratio) was then determined as an index of small intestinal permeability. Participants were also asked to report any adverse gastrointestinal symptoms.

### 3.10. Nutrition

Participants were provided with a copy of, and were instructed to eat according to, the 12345+ Food and Nutrition Plan [23]. Participants were required to complete a food diary every eighth day during the study period starting on Study Day 4. This ensured that all days of the week had been surveyed by the end of the study period. Dietary intakes were subsequently analysed (SERVE Dietary Analysis Software, M.H. Williams, Adelaide, South Australia) to determine average daily energy and macronutrient (*i.e.*, carbohydrate, fat and protein) intakes.

### 3.11. Statistical Procedures

Univariate analysis of variance (ANOVA) was used to compare group means at baseline and dietary intakes. To determine the effects of the treatments, time of measurement and their interactions on the dependant variables, univariate analysis of variance (ANOVA) with repeated measures was used. One factor was the treatment group (*i.e.*, BC, WP or CON) and the other (with repeated measures) was the time of measurement of the dependant variable. There was a significant difference in the lactulose:rhamnose ratio between groups at baseline, so changes in this parameter were assessed using analysis of covariance (ANCOVA) with the baseline measures being used as the covariate. Where ANOVA or ANCOVA showed a statistically significant main effect, pair-wise comparisons were made using post-hoc analysis for least significant differences. Linear regression analysis was used to determine relationships between variables where appropriate. Statistical significance was set at *P* < 0.05. Unless otherwise stated, all data values cited in the text and shown in tables and figures represent means ± standard error.

## 4. Conclusions

The present study indicated that the consumption of bovine colostrum during eight weeks of endurance exercise training increased intestinal permeability compared with whey protein and a control. Future studies should investigate the potential for bovine colostrum to increase the transport of large molecules across the intestinal epithelium.
